# Inter and intra cultural variations of millet (*Pennisetum glaucum* (L.) R. Br) uses in Niger (West Africa)

**DOI:** 10.1186/s13002-019-0321-4

**Published:** 2019-08-13

**Authors:** Hamadou Moussa, Valentin Kindomihou, Thierry D. Houehanou, Idrissa Soumana, Oumarou Souleymane, Mahamadou Chaibou

**Affiliations:** 1 Institut National de la Recherche Agronomique du Niger, BP 429, Niamey, Niger; 2 Laboratoire d’Ecologie Appliquée, Facultés des Sciences Agronomiques, Université d’Abomey-Calavi, 01 BP 526, Cotonou, Benin; 3 Laboratoire d’Ecologie, de Botanique et de Biologie Végétale, Faculté d’Agronomie, Université de Parakou, 03 BP 125, Parakou, Benin; 4 Institut National de la Recherche Agronomique du Niger, BP 429, Niamey, Niger; 5 Institut National de la Recherche Agronomique du Niger, BP 429, Niamey, Niger; 6 Département des Productions Animales, Faculté d’Agronomie, Université Abdou Moumouni de Niamey, BP 10 960, Niamey, Niger

**Keywords:** *Pennisetum glaucum*, Uses, Organs, Ethnic group, Niger

## Abstract

**Background:**

An ethnobotanical study was conducted in the eight regions of Niger to identify local knowledge variation of millet (*Pennisetum glaucum* (L.) R. Br) uses. In fact, the level of individual knowledge can be affected by many factors such as gender, age, ethnicity, occupation, religious and cultural beliefs, etc. This study documented indigenous knowledge of millet uses in Niger and aimed specifically to (i) identify the different types of millet organ uses and (ii) assess the variation of local knowledge of millet uses along with ethnicity, occupation, and age.

**Methods:**

The data were collected in 32 major millet-producing villages in Niger through individual semi-structured interviews and focus group discussions. About 508 individuals from 5 ethnic groups were interviewed. The assessment of the knowledge was performed by calculating five ethnobotanical indices such as the number of reported uses by parts of the plant (RU), the use-value of the parts of the plant (PPV), the specific use-value (SU), the intraspecific use-value (IUV), and the relative frequency of citations (FRC). Data were analyzed using descriptive, univariate, and multivariate statistical analyses.

**Results:**

The results indicated a significant variation in uses across ethnic groups (*H* = 38.14, *P* = 0.000) and socio-occupational categories (*H* = 6.80, *P* = 0.033). The *Hausa*, *Kanuri*, and *Zarma-Sonhrai* ethnic groups, farmers were the largest users of the species. Dietary (51.40%) and forage (40.35%) were the most reported uses. The most commonly used parts of the plant were the stubble (74.92%) and grains (73.68%).

**Conclusions:**

The study showed the importance of *P. glaucum* in the daily life of local people. It also confirmed the uneven distribution of indigenous knowledge of millet uses in Niger due to social factors. Now, the challenge is how to incorporate these social differences in knowledge of millet uses in view to sustainable management and conservation of local genetic resources of millet. Finally, this work could be an important decision-making tool for future millet valuing.

## Introduction

Millet (*Pennisetum glaucum* (L.) R. Br) is a staple food crop in arid and semi-arid areas of Asia and Africa and remains one of the main sources of energy, protein, vitamins, and minerals for millions among the poorest people in these regions. This cereal is generally grown for grains, used in human and animal diet, and also for stubble used as fodder and silage [1]. In addition to the dietary and forage use of millet, different parts of the plant are commonly used for multiple services including the treatment of various human and animal diseases [2, 3], soil fertilization, and handicrafts [4, 5]. Furthermore, as a result of climate change and population pressure, millet is increasingly being exploited as forage or a dual-purpose crop (grain and fodder) in order to ensure the food security of livestock [6, 7]. This new trend towards the valuation of millet in animal food is not without consequences on the food security of the human local populations. Therefore, an ethnobotanical study appears to be a good approach in this area to understand the use as well as the sociocultural and economic perceptions of local populations about this crop [8–10].

Ethnobotany is a science that is related to several disciplines such as biodiversity conservation, conservation genetics, ethno-pharmacology, food technology, ecology, etc. [11]. The ethnobotanical assessment of millet would be then indispensable for its valuation, sustainable management, and conservation. This study documented indigenous knowledge of millet uses by ethnic groups in Niger. Past ethnobotanical studies in the West African Sahel have focused on wild woody and herbaceous plant species [12, 13]. But this study was focused on a crop such as millet, given its importance as a major cereal for humans and as an additional source of forage for animals in Niger. However, little known work has been conducted on the ethnobotanical use of millet despite being considered as the staple food crop for local populations in the arid and semi-arid areas of the world [14–16]. The objectives of our study were to document the endogenous knowledge of millet uses in Niger and to assess the effects of ethnicity, occupation, and age on botanical knowledge. Indeed, Indigenous knowledge is often unevenly distributed among those factors [13]. Moreover, the level of individual knowledge of native plant species can be affected by many factors such as sex, age, ethnicity, occupation, religious and cultural beliefs, abundance, and the usefulness of the species [13, 17]. In addition, research conducted in the West African Sahel reported that the *Fulani*, *Kel Tamashek*, *Bellah*, and *Maure* groups were the main major livestock-rearing groups, while the farmers were mainly from the *Bambara*, *Hausa*, *Djerma*, *Gourmantche*, *Mossi*, and *Soninke* [13, 18]. Nowadays, professional specialization according to ethnic criteria is becoming increasingly blurred in the region [13, 18]. Nevertheless, pastoral groups usually know more about livestock than farmer groups and vice versa. Robert et al. [19] also reported that producers’ choice of millet varieties is generally based on agro-morphological traits, phenological, or organoleptic characteristics. Furthermore, the preservation of the cultural identity of a community requires knowledge to be passed on from generation to generation [13]. Age therefore has an impact on the knowledge of plants within ethnic groups [13, 20]. In this study, we tested three hypotheses. First, ethnicity affects knowledge about the uses of millet organs, so that farmers (*Zarma-Sonhrai*, *Hausa*, *Kanuri*, *Gurmantche*) tend to know the uses of millet better than pastoralists (*Fulani*, *Tuareg*, *Tubu*). Secondly, the socio-professional category also influences the knowledge of uses of millet organs, so that ethnic groups such as *Zarma-Sonhra*i, *Hausa*, *Kanuri* and *Gurmantche* (farmers) tend to know the uses of the millet organs better than *Fulani*, *Tuareg*, *Tubu* (pastoralists). And thirdly, there is a positive correlation between knowledge of millet organ use and age, that is, older people are more familiar with millet uses than younger people.

## Materials and methods

### Area of the study

**Fig. 1.**
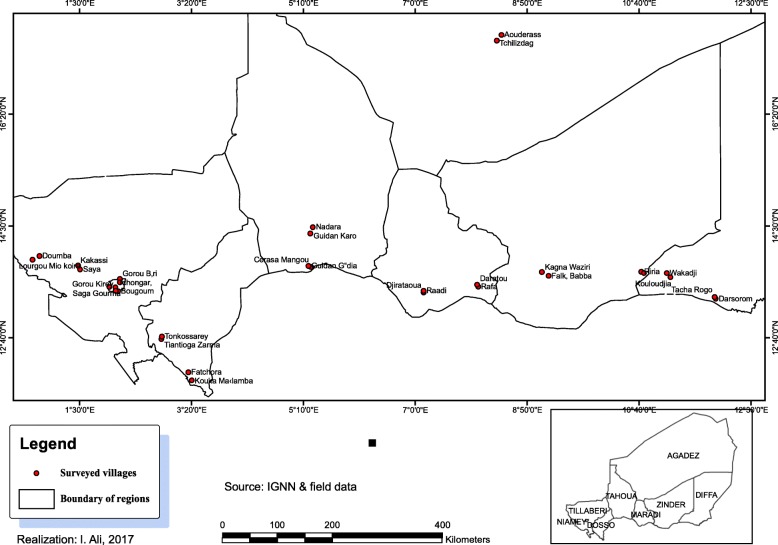
Location of the surveyed villages

This study was carried out in the eight regions of Niger Republic (Fig. 1). Niger is located in West Africa, between latitudes 11° 37′ and 23° 23′ N and longitudes 0° and 16° E. It is located 700 km from the Gulf of Guinea, 1900 km from the East Atlantic coast, approximately 1200 km from the Atlantic coast to the south and north of the Mediterranean sea [21]. It covers an area of 1,267,000 km^2^ and is divided into 8 regions (Fig. 1), 36 provinces, and 265 municipalities (52 urban and 213 rural). Niger is inhabited by eight ethnic groups that are mainly situated in the following regions: *Hausa*: Maradi, Tahoua, Zinder, and Dosso regions; *Zarma-Sonhrai*: regions of Tillabéri, Dosso, and Niamey; *Tuareg*: regions of Agadez, Tahoua; *Fulani*: regions of Niamey, Dosso, Maradi, Tahoua, Diffa, Tillabéri, and Zinder; *Kanuri*: regions of Diffa and Zinder; *Tubu*: regions of Diffa and Zinder; *Arabs*: regions of Tahoua, Diffa, Agadez and Zinder; *Gurmantche*: Tillabéri region [22].

The estimated population of Niger is 19,865,068 inhabitants. It is a relatively young population with about 58.4% under 18 years old [22]. Niger’s economy is mainly based on farming, trade, and handicrafts. The main cultivated species are cereals (millet, sorghum, rice, maize, fonio) and cash crops (cowpea, nutgrass, groundnut, sesame, sorrel, tiger nut, and cotton) [23].

Livestock is one of the most important riches in Niger. The national population, estimated at 14,467,087 UBT in 2012, is composed of cattle, sheep, goats, camels, horses, and donkeys [24]. The population of Niger is mostly rural (almost 83.8%) and its income derives mainly from the exploitation of natural resources [25]. In almost the regions, farming is the first contributors to household incomes [26].

The terrain is characterized by a large peneplain with an average altitude of 500 m with depressions and elevated points especially in the northern part.

The altitude increases from the south to the north where the mountainous areas (Aïr, Termit) exceed 900 m. The soil textures range from sandy to clay-sandy, poor in nutrients, and organic matter. Arable soils are of 80% dunes and 15–20% are moderately composed of clay and hydromorphic soils [24]. The climate is a semi-arid tropical type, characterized by two seasons: a dry season from October to May and a rainy season from June to September. During the dry season, the average temperature fluctuates between 18.1 and 33.1 °C. However, during the rainy season, this temperature varies between 28.1 and 31.7 °C [25].

### Sampling and data collection

The data were collected in 32 major millet production villages in Niger from January to February 2016. The collection was performed via individual semi-structured interviews and focus group discussions (groups of two to 15 people) in selected locations based on stratified sampling. Three levels of stratification were selected: socio-cultural or ethnic groups (first level), the best production provinces of millet (second level) and villages (third level). A total of 32 villages were surveyed on the use of millet. Participants in the surveys were randomly selected based on the methods of Uprety et al. [27]. Interviews were conducted in the most commonly spoken local languages in Niger (*Hausa* and *Zarma*) but translators intervened when the interlocutor did not speak any of the two languages. These surveys were supplemented by the collection of seeds from local farmers when they were available.

### Data analysis

The assessment of the knowledge was conducted using the computations of the ethnobotanical indices of the plant as defined by Gomez-Beloz [28] and used for species-specific studies [9, 29, 30]. A total of five ethnobotanical indices were computed: the reported use (RU), the plant part value (PPV), the specific reported use (SU), the intraspecific use-value (IUV) and the relative citation frequency (FRC).

The reported use (RU) is the total number of uses reported for the plant. It is represented by the number of uses reported for each plant part:

$$ RU={\sum}_{i=1}^nR{U}_{\mathrm{plantpart}} $$

The plant part value (PPV) is equal to the ratio between the total number of total uses reported for each plant part and the total number of the reported uses for the plant:

$$ \mathrm{PPV}={\mathrm{RU}}_{\mathrm{plant}\ \mathrm{part}}/\mathrm{RU} $$

The most often used parts of the species by the respondents from an ethnic group are those having high values of PPV.

The specific reported use (SU) is the use as described by the respondents. It refers to the number of times a specific reported use is mentioned by the respondents from an ethnic group:

$$ SU={\sum}_{i=\mathrm{o}}^n{c}_i $$

The intraspecific use-value (IUV) is the ratio of the specific reported use to the reported use for the plant part. It helps to identify for a specific plant part, the most reported specific uses by the respondents from an ethnic group:

$$ \mathrm{IUV}={\mathrm{SU}}_{\mathrm{plant}\ \mathrm{part}}/{\mathrm{RU}}_{\mathrm{plant}\ \mathrm{part}} $$

The relative frequency of citation (FRC) for an organ (or use) was adapted to the formula of Ladoh-Yemeda et al. [31] and is calculated as follows:

$$ FRC=\frac{\overline{\mathrm{N}}\mathrm{c}}{Ne}\mathrm{x}100 $$

$\overline{\mathrm{N}}\mathrm{c}$ refers to the number of times that a given organ (use) has been cited for a specified purpose and does not have the (social) factor in question.

The Kruskal-Wallis test [32] was performed to test the dependence of the relative frequency of quotations according to the ethnic group, the age class, and the profession. The three social factors were combined by defining 36 sub-groups. Thus, the relative frequency matrices of the specific uses of the *P. glaucum* parts were subjected to a principal component analysis (PCA) using the software R [33] with the constituted sub-groups. In addition, for the interpretation of a given point (social factor or specific use) on an axis of the PCA, two criteria have been retained [34, 35]:

- A good contribution (CTR) such as CTR ≥ 100/*n* (*n* = number of individuals/variables);
- A good quality of representation (COS2) on the axis such as COS2 ≥ 0.3.

### Socio-economic profiles of respondents

A total of 508 individuals across 5 ethnic groups were surveyed (Table 1). Respondents were divided into ethnic group, age group, and socio-occupational category. Hence, six sub-groups were defined for each ethnic group: young (Je), adult (Ad), old (Vx), farmers (Ag), farmers-pastoralists (Aél), and *Fact* (*Fonctionnaires-artisans-commerçants-transporteurs* in French, Civil servants-craftsmen-traders-transporters in English). Similarly, three sub-groups were defined for each socio-occupational category: young (Je), adult (Ad), and old (Vx). Thus, 39 sub-groups (5 ethnic groups × 6 sub-groups + 3 socio-occupational categories × 3 sub-groups) are expected, but due to the absence of certain subgroups, only 36 sub-groups were taken into account (Tables 1, 2, and 3).

**Table 1:** Ethnic and age groups samples

	*Hausa*	*Kanuri*	*Fulani*	*Tuareg*	*Zarma-Sonhrai*	Total
Young people (ages < 40)	57	38	7	7	45	154
Adults (40 ≤ ages < 60 years	94	33	30	21	80	258
Old (60 years ≤ ages)	35	8	17	7	29	96
Total	186	79	54	35	154	508

**Table 2:** Ethnic group and socio-occupational category samples

	*Hausa*	*Kanuri*	*Fulani*	*Tuareg*	*Zarma-Sonhrai*	Total
Farmers	170	73	36	35	144	458
Farmer-herders	2	5	14	–	2	23
Fact	14	1	4	–	8	27
Total	186	79	54	35	154	508

**Table 3:** Age group and socio-occupational category samples

	Farmers	Farmer-herders	Fact	Total
Young people (ages < 40)	138	9	8	155
Adults (40 ≤ ages < 60 years	235	14	10	259
Old (60 years ≤ ages)	86	-	8	94
Total	459	23	26	508

## Results

### Types of use

**Fig. 2:**
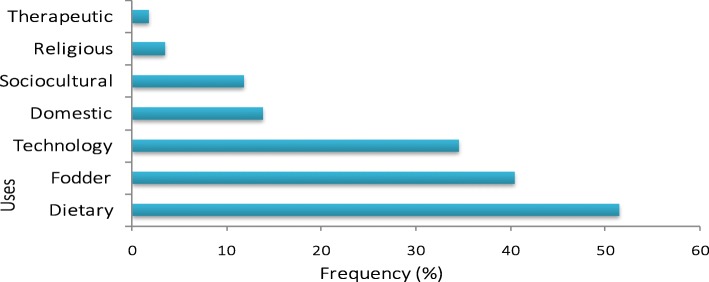
Relative citation frequencies of the millet different uses

Multiple parts of *P. glaucum* were used for various purposes by the different ethnic groups in Niger. There have been recorded seven types of use (Fig. 2), which are the dietary use, the therapeutic use, the technological use, the socio-cultural use, the domestic use, the religious use, and forage. Food has been the highest reported use (51.40%), followed by forage use (40.35%) while therapeutic use has been the least cited (1.69%).

### Parts used

**Fig. 3:**
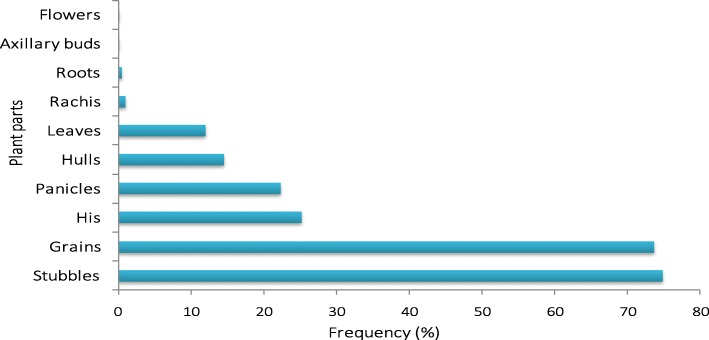
Relative citation frequencies of the different parts used of the millet

All the millet parts have been used from the leaves to the roots. We have 10 different parts used (Fig. 3). The most-reported parts were the stubble and the grains with respective relative frequencies of 74.92 % and 73.68%. The axillary buds and the flowers were the least listed parts with relative citation frequencies of 0.11% and 0.08% respectively.

### Millet use variation based on social factors

Significant difference existed between *Hausa*, *Kanuri*, *Peulh*, *Tuareg*, and *Zarma-Sonhrai* (*H* = 38.14, *P* = 0.000). Significant difference was also observed between farmers, agro-herders, and *Fact* (*H* = 6.80, *P* = 0.033) in terms of *P. glaucum* parts use. However, there is no significant difference between adults, young, and old (*H* = 2.82, *P* = 0.244) in the use of *P. glaucum* parts.

#### Use variation based on ethnic and age groups

**Fig. 4:**
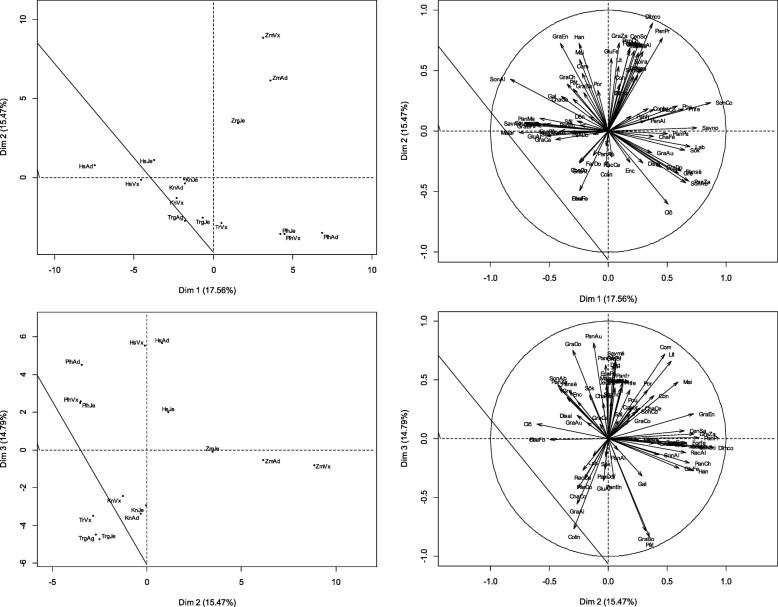
Factorial map of the PCA describing the relationships between the specific uses of millet and the age-ethnic group factor. Alivo = travel food; Allfe = fire lighter; Attbo = Attache boot; Boira = refreshing drink; CenAb = ash for watering cattle; Cenpa = ash for wound dressing; CenSa = ash sauce; CenSo = ash for soumbala; ChaCe = ash stubble for cooking; ChaCo = stubble as compost; ChaFe = stubble as fertilizer; ChaFo = forage stubble; Clô = closing; Colin = guest snack; Com = fuel; Conbr = manufacture brick; Conre = ash for meal conservation; Con = construction; Deg = Dégué; Déspa = parcel desalinization; Dîmco = customary tithe; Disal = food discrimination according to sex; Don = Donu; Enc = Enclos; EpaPa = thickener paste; FabOr = manufacture oreillets; FarDo = flour doum; FeuFo = fodder sheets; Filcu = culinary filter; Forfe = fortifying for breastfeeding woman; Gal = galette; GluAl = glumes feed cattle; GluCa = glumes carbonization wood charcoal; GluCo = glumes compost; GluFe = fertilizer glumes; GluPo = glumes pottery; GraAl = grains livestock feed; GraAu = grains aumone; GraBi = bita grains; GraBo = grains for porridge; GraCa = grains for engagement gifts; GraCh = grains for charity; GraCo = couscous grains; GraDo = Grains for late harvest donation ; GraEn = grains for social assistance; GraMa = grains for gift to marabouts; GraZa = grains for zakkat; Gre = grenier; Han = hangar; Jeuma = provision for bride; Grains; Lab = labdourou; Bed = beds; May = house; Malar =clay mixing; PanAl = panicle for livestock feed; Panfr = fresh pan for grillade; Pansè = dry pan for grilling; PanAu = Panicles for Alms; PanCh = panicles for charity; PanDo =panicles for donation in late harvest; PanEn = panicles for social assistance; PanMa = panicles for donation to marabouts; PanPa = panicles for donation to parents; PanPr = panicles for provision for primiparous women; PanZa = panicles for zakkat; Pât = paste; Por = portal; Pou = henhouse; Prife = grains for provision for primiparous woman; RacAl = spoiled for livestock feed; Rack = spit in ash for cooking; Sal = sala; Savmé = medical soap; Savno = black soap; Sék = Sékos; Sôk = Sôkou; SonAb = sound for livestock watering; SonAl = sound for livestock feed; SonBo = sound for boiled; SonCo = sound for couscous; SosKo = Sosso Komandi; Sou = souroundou; SubNa = ash as a substitute for natron; Was = Wassalé; Zor = Zori. HsAd = hausa adult; HsJe = hausa youth; HsVx = hausa old; KnAd = adult kanuri; KnJe = young kanuri; KnVx = old kanuri; PlhAd = adult fulani; PlhJe = young fulani; PlhVx = old fulani; TrgAd = adult Tuareg; TrgJe = young tuareg; TrVx = old tuareg; ZmAd = adult zarma-sonhrai; ZmJe = zarma-sonhrai young; ZmVx = old zarma-sonhrai

The PCA showed that the first three axes explained 62.8% of the variation observed among the various forms of the species use (Fig. 4). The specific uses of *P. glaucum* were known by all ethnic groups. Nevertheless, the relative frequencies of citations of *P. glaucum* use varied significantly from one sub-group to another based on the combined factor “ethnic group-age group” (*H* = 37.86, *P* = 0.001). Axis 1 contrasted adult and old *Hausa* with adult *Zarma-Sonhrai*. The first group was known for the use of panicles to donate to parents (0.062 ≤ IUV ≤ 0.115), the consumption of grains processed into traditional foods such as *labdourou* (the *dônou* that has not been baked), or its use to “accompany” primiparous women on maternity leave.

It was also noticed among old and adult *Hausa* the use of ash (from stubble) in the manufacturing of black soap. The *Zarma-Sonhrai* adults were characterized by the use of stubble ash in livestock watering (IUV = 0.775) or as medical soap, in the processing of grains into special dishes such as *sosso komandi* (natron porridge), *bita* (kind of very thick porridge), or *souroundou* (equivalent of millet-type rice dish). It was also worth noting that among *Zarma-Sonhrai* adults, the use of glumes in pottery, the composting of agricultural residues, or the carbonization of wood in charcoal, as well as in the mixing of building clay (IUV = 0.382) or for manufacturing pillows. The Z*arma-Sonhrai* adults additionally offered panicles to religious leaders and used the bran (IUV = 0.796) and *zori* (liquid from the washing of milled cereal grains) in the animal feeding. Axis 2 compares elderly *Fulani* and *Zarma-Sonhrai*. Elderly *Fulani* mainly used the stubbles and the millet bran. The stubbles were used in the fencing and while the bran was used in animal watering (IUV = 0.3). The elderly *Zarma-Sonhrai* used all the parts of millet. Thus, the grains were processed into fortifying diets for lactating women or make *wassalé* (kind of semolina grilled with butter or oil). Elderly *Zarma-Sonhrai* also used the grain as gifts to religious leaders to pay off the *zakat* (alms given at the end of the month of *Ramadan*) or socially as a way of mutual aid (IUV = 0.108). Elderly *Zarma-Sonhrai* also used the stubble in building houses (IUV = 0.130) or to make cooking fire. The use of the stubble ashes was mentioned by this category of people to give a special taste to sauces or to accelerate the cooking or to make *soumbala* (mustard made from sorrel grains). The elderly *Zarma-Sonhrai* finally made use of stubble as fodder and panicles to accompany primiparous women on maternity leave in their families during the usual 40 days. The axis 3 contrasted elderly *Fulani* with adult *Kanuri* and young and adult *Tuareg*. Four millet parts such as the grains, the panicles, the stubble, and the bran were used by *Fulani* adults. The stubbles were used to make fire, bed, or medicine with ashes (medical soap and sticky-plaster), while the bran was used as a drink for animals (IUV = 0.745). The panicles were used to give out *zakat* (compulsory alms given at the end of the harvest), for the alms or given as gifts to relatives. The grains were processed into a local beverage, which is a mixture of millet flour balls dissolved into milk or yogurt. This newly obtained mixture is very popular with all ethnic groups in Niger. Its name varies from one ethnic group to another. Thus, it is called *dônou* by the *Zarma-Sonhrai*, *furah* by the *Hausa*, *chobbal* by the *Peulh*, *tidda* by the *Tuareg*. *Kanuri* adults, *Tuareg* young and adults were characterized by the exclusive valuing of the millet grains in the human and animal diet. The grains have essentially been processed to make some porridge, paste during socio-cultural ceremonies, or processed into simple fodder or mixed with other animal foods.

#### Use variation based on the occupation and ethnic group

**Fig. 5:**
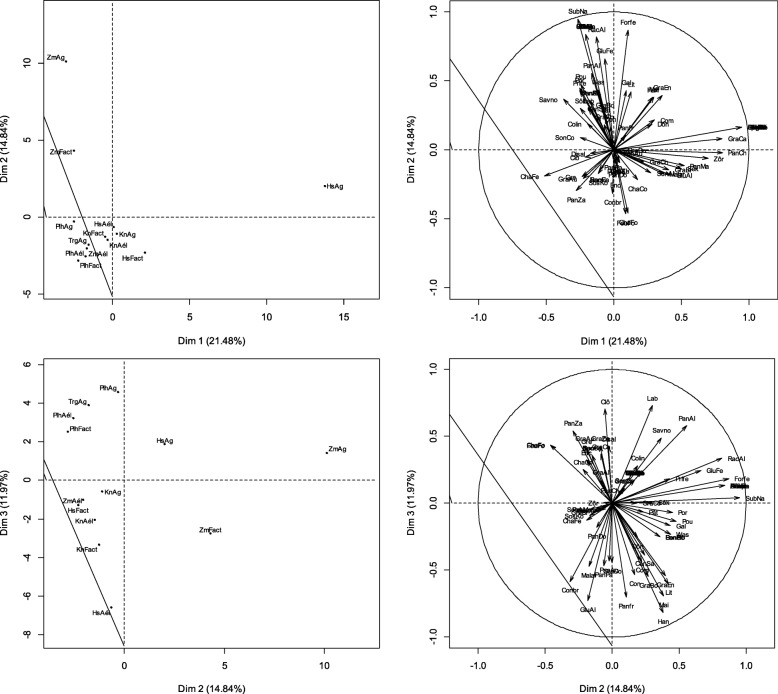
Factorial maps of the PCA describing the relationships between the specific uses of millet and the occupation-ethnic group factor. Note: HsAg = hausa farmer; HsAel = hausa agro-herder; HsFact = hausa others; KnAg = kanuri farmer; KnAel = kanuri agro-herder; KnFact = kanuri others; PlhAg = fulani farmer; PlhAel = fulani agro-herder; PlhFact = fulani others; TrgAg = tuareg farmer; ZmAg = zarma-sonhrai farmer; ZmAel = zarma-sonhrai agro-herder; ZmFact = zarma-sonhrai others

The PCA revealed that the three first axes explained the 67.44% of the variance observed between the different types of uses of the species (Fig. 5). The specific uses of the *P. glaucum* were known by all the socio-occupational categories. Nevertheless, the relative frequencies of citations of *P. glaucum* use varied significantly from one sub-group to another according to the combined factor “ethnic group-occupational category” (*H* = 42.92, *P* = 0.000). Axis 1 singled out *Hausa* farmers who were characterized by the use of the stubbles as fertilizers in fields, the use of the millet bran to thicken the paste, the processing of the millet grain into local foods such as *chokkou (or sokou)*, *dèguè*, and *sâlâ* (a variant of millet cake) or as presents given to relatives and neighbors as well as giving panicles as simple presents or as a way of mutually helping one another in the society.

Axis 2 isolated the *Zarma-Sonhrai* characterized by the use of grain-derived foods, panicles, stubble (ash substituting natron), bran (refreshing drink), and rachis (cattle feed (IUV = 1)). Axis 3 contrasted the *Fulani* farmers with the *Hausa* farmers and herders and *Kanuri Fact*. The *Fulani* farmers used the grains as *labdourou*, panicles as cattle feed or to give out *zakat* and stubble as fence. The *Hausa* farmers and herders and *Kanuri Fact* used the glumes as cattle feed (0.5 ≤ IUV ≤ 0.6) or to make bricks (0.2 ≤ IUV ≤ 0.5). The *Hausa* farmers and herders and *Kanuri Fact* also used the stubble for the construction of beds and houses, the grains to make porridge and actions of solidarity. Finally, they ate the fresh panicles grilled on embers.

#### Use variation based on occupation and age group

**Fig. 6:**
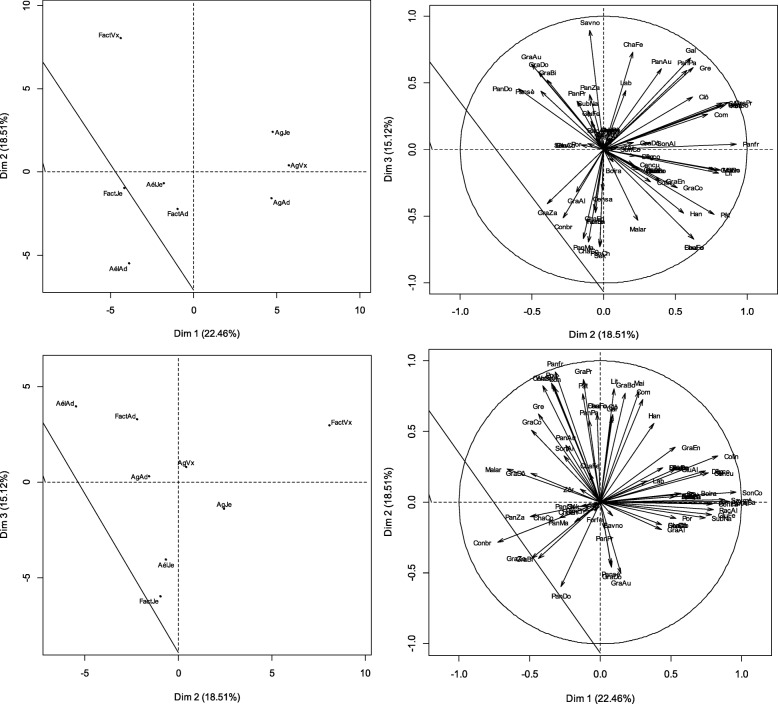
Factorial maps of the PCA describing the relationships between the specific uses of millet and the occupation-age factor. Note: AgAd = adult farmer; AgJe = young farmer; AgVx = old farmer; AelAd = agro-herder adult; AelJe = young Agro-herder; AelVx = Agro-herder old; FactAd = other adults; FactJe = other young people; FactVx = other old people

The PCA indicated that the three first axes explained 62.21% of the total variance observed between the different types of use of the species (Fig. 6).

All age groups knew the specific uses of *P. glaucum*. Nevertheless, the relative frequency citations of *P. glaucum* significantly varied from one subgroup to another along with the combined factor “age group-socio-occupational categories” (*H* = 24.83, *P* = 0.001). Axis 1 isolated the group of farmers (all ages) who essentially used five millet parts. The grains were processed into various foods: snack for visitors, donation as a mutual aid (0.107 ≤ IUV ≤ 0.141), gifts to brides, preparation of dishes such as *souroundou* or *dèguè* (made with couscous from millet and yogurt or curdled milk). The bran was processed into a refreshing drink, as a dough thickener or as black couscous. The stubbles were known for being used to light fires, being incinerated for ash and used in cooking, sticking-plaster for wounds, as an ingredient in sauces or making medical soap. The panicles were used as cattle feed and in the payment of customary tithe to landowners. Finally, panicles have been valued by farmers as animal’s food. Axis 2 compared adult herders and adult farmers to the old *Facts*. The adult breeders and adult farmers were more interested in using millet grains to give alms. The old *Facts* were more interested in the use of leaves as fodder (IUV = 1), stubble in various constructions, as fuel or as fodder and grains as porridge or couscous. Axis 3 isolated young *Fact*; this group was interested in the using of stubble in domestic work (making thatches) and panicles in charitable actions. However, this group was particularly not uninterested in dishes made from processed millet grains.

## Discussion

This study revealed that the level of use of the millet parts varies depending on ethnicity and profession. Previous studies conducted on cassava varietal diversity in the northwest Amazon area in Brazil also reported a strong correlation between varietal diversity and cultural identity within local ethnic groups [36]. Results showed that the ethnic groups that mostly use the *P. glaucum* organs in Niger were *Hausa* and *Zarma-Sonhrai*. The Nigerien ethnic groups such as *Zarma-Sonhrai*, *Hausa*, *Kanuri*, and *Gourmantche* are essentially farmers. Consequently, farming is their main activity. On the other hand, ethnic groups such as *Fulani*, *Tuareg*, *Tubu*, and *Arab* devoted almost exclusively to breeding and were therefore considered traditionally as “pastoralists” or “nomads” [37]. Therefore, it is quite normal that the *Zarma-Sonhrai* and the *Hausa* appeared as the greatest users of *P glaucum* organs specifically in this study. These results confirm the differentiation of knowledge along ethnic groups in our study. These results are very closed to those of Jika et al. [38], claiming that millet has a higher symbolic value in the rural communities of *Zarma-Sonhrai*, *Hausa*, and *Kanuri*, which represents a strong social barrier in the dissemination of seeds between these ethnolinguistic groups [38]. Furthermore, similar studies conducted on other species with important socio-economic value on a regional scale confirm these observations [29, 30]. Ethnicity, therefore, remains one of the major factors of difference in use and knowledge of the plants among communities [20].

A significant difference in term of knowledge level of use of millet organs was revealed among socio-occupational categories. In fact, farmers knew more about plant growing and conservation because of their close dependence on it as food crops or its other related uses. This explains the particularity of farmers in the abundant and diversified uses of different organs of *P. glaucum* in contrast with herders and agro-pastoralists who use it little and specifically. These results confirm the assumption of knowledge dependent on the socio-professional category. These results corroborated the findings of Jika et al. [38] who found out that there was a strong attachment to certain species among Sahelian farmers to their own local varieties of millet in western Lake Chad area. According to the same authors, the attachment of farmers to certain millet varieties can be linked not only to symbolic and aesthetic considerations but also to the way in which these varieties match the different expected uses [38]. In addition, Robert et al. [39] reported from southern Niger, the farmer’s preference to grow their own local varieties because of their adaptation to their cropping systems. For example, the seed of local varieties acquired from outside sources (NGO; market) is mainly consumed but rarely sown [39]. Moreover, our results revealed that the elders of *Fact* group showed a particular interest in the use of leaves and stubble of *P. glaucum* as fodder. This behavior is explained primarily by their status, which allowed them to pursue other income-generating activities such as cattle breeding. Most of these actors live in urban areas where animal feeding costs are the highest [40]. But it turns out that millet-based forage is one of the most economically accessible forage for farmers [41]. This could well justify the special interest shown by the old *Fact* for millet fodder.

No significant difference was observed in the use of the organs of *P. glaucum* according to age. Our third hypothesis is therefore not completely verified in this study. Nevertheless, significant differences were observed in the uses of *P. glaucum* parts when the age factor was associated with other factors such as ethnicity or occupation of the respondent. In other words, there was no variation in the use of the organs of *P. glaucum* between young people, adults, and old, when age factor was taken aside. However, variations in the use of *P. glaucum* organs were observed when the analysis was performed with combined factors: age-ethnicity and age-profession. Thus, we observed that young *Tuareg*, young farmers, and young *Fact* also used *P. glaucum* organs. Young farmers’ knowledge on millet use is obviously natural as inherited from their parents. Indeed, some authors support the idea that knowledge is transmitted from a generation to another within the same ethnic group [20]. As far as the young *Fact* are concerned, their knowledge of the use of millet resulted from their greater consumption of new millet products that were coming from the agri-food industry technologies. Indeed, it is nowadays easy to find on supermarket shelves of urban centers in Niger various local millet grains-based products, i.e., *dèguè*, lumps, oilcakes, enriched powder, developed by local farmer organizations. Similarly, millet fodder is processed into products in animal feed with the advent of new grinding and chopping machines in the Sahel [5]. These results are confirmed by Kébenzikato et al. [20] who found out that people over 75 years old had a greater knowledge of the uses of *Adansonia digitata* in Togo. Thus, Ayantunde et al. [13] showed that the age group above 50 years old knew more than that between 25 and 50 years old.

## Conclusion

This study highlighted 10 different parts used of *P. glaucum*, which were identified and used differently into five ethnic communities in Niger. The uses of grains and panicles of this cereal are very common and these products are well consumed by all surveyed ethnic groups. The *Hausa*, *Kanuri*, and *Zarma-Sonhrai* ethnic groups and farmers are the largest users of the species. The elderly *Fact* group was the most users of millet stubbles and leaves as fodder. This ethnobotanical survey based on individual interviews and focus groups revealed the importance of *P. glaucum* in the life of local people. This method that solicits the memory of respondents could obviously cause bias related to the personal assessment of the respondent. However, this method is widely used in ethnobotany by many authors and has the advantage of showing rather conclusive results most of the time. Results from this study confirmed the uneven distribution of indigenous knowledge of millet uses in Niger due to social factors. But the challenge is how to incorporate these social differences in knowledge of millet uses in view to sustainable management and conservation of local genetic resources of millet. As the uses of millet organs are poorly documented in Niger, this study provides a broad overview of the uses made of millet organs following ethnic groups and socio-professional categories. Therefore, this work could be an important decision-making tool for future millet valorization studies as forage or dual-purpose crop. Moreover, the study gives some insights into the importance of bio cultural diversity conservation in Niger. Because of knowledge variation among different ethnic groups, culture of those groups must receive an important consideration for conservation.

## Data Availability

The datasets used and/or analyzed in the current study are available from the corresponding author on reasonable request.

## Abbreviations

FRC: Relative citation frequency
IUV: Intraspecific use value
PCA: Principal component analysis
PPV: Plant part value
RU: Reported use
SU: Specific reported use
