# Correction to: Inter and intra cultural variations of millet (*Pennisetum glaucum* (L.) R. Br) uses in Niger (West Africa)

**DOI:** 10.1186/s13002-019-0324-1

**Published:** 2019-09-09

**Authors:** Hamadou Moussa, Valentin Kindomihou, Thierry D. Houehanou, Idrissa Soumana, Oumarou Souleymane, Mahamadou Chaibou

**Affiliations:** 1 Institut National de la Recherche Agronomique du Niger, BP 429 Niamey, Niger; 2 Laboratoire d’Ecologie Appliquée, Facultés des Sciences Agronomiques, Université d’Abomey-Calavi, 01 BP 526 Cotonou, Benin; 3 Laboratoire d’Ecologie, de Botanique et de Biologie Végétale, Faculté d’Agronomie, Université de Parakou, 03 BP 125 Parakou, Benin; 4 Institut National de la Recherche Agronomique du Niger, BP 429 Niamey, Niger; 5 Institut National de la Recherche Agronomique du Niger, BP 429 Niamey, Niger; 6 Département des Productions Animales, Faculté d’Agronomie, Université Abdou Moumouni de Niamey, BP 10_960 Niamey, Niger

**Correction to: J Ethnobiol Ethnomed**

**https://doi.org/10.1186/s13002-019-0321-4**

Please note that following publication of the original article [1], Figs. 4, 5 and 6 in the article have been updated to remove oblique lines that were erroneously rendered in the figures.

The figures are now correct in the original article.For reference, please also find the (corrected) figures below:

**Fig. 4:**
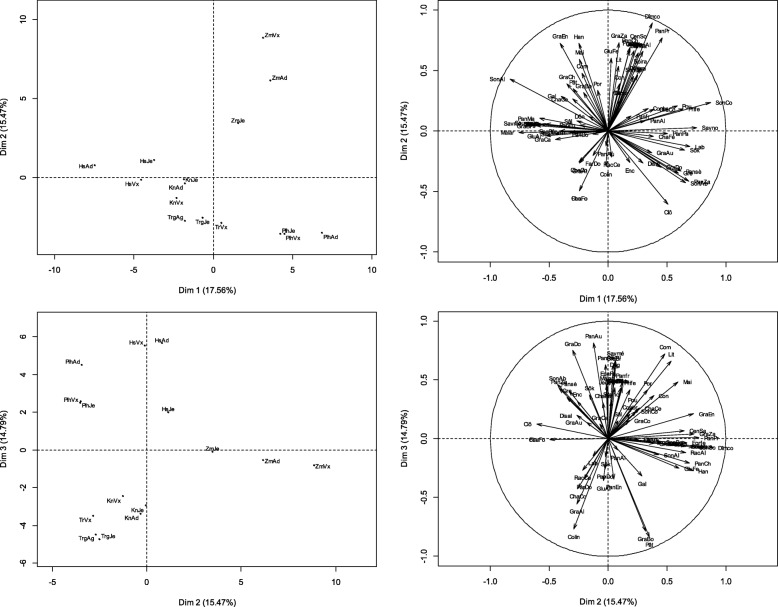
Factorial map of the PCA describing the relationships between the specific uses of millet and the age-ethnic group factor. Alivo = travel food; Allfe = fire lighter; Attbo = Attache boot; Boira = refreshing drink; CenAb = ash for watering cattle; Cenpa = ash for wound dressing; CenSa = ash sauce; CenSo = ash for soumbala; ChaCe = ash stubble for cooking; ChaCo = stubble as compost; ChaFe = stubble as fertilizer; ChaFo = forage stubble; Clô = closing; Colin = guest snack; Com = fuel; Conbr = manufacture brick; Conre = ash for meal conservation; Con = construction; Deg = Dégué; Déspa = parcel desalinization; Dîmco = customary tithe; Disal = food discrimination according to sex; Don = Donu; Enc = Enclos; EpaPa = thickener paste; FabOr = manufacture oreillets; FarDo = flour doum; FeuFo = fodder sheets; Filcu = culinary filter; Forfe = fortifying for breastfeeding woman; Gal = galette; GluAl = glumes feed cattle; GluCa = glumes carbonization wood charcoal; GluCo = glumes compost; GluFe = fertilizer glumes; GluPo = glumes pottery; GraAl = grains livestock feed; GraAu = grains aumone; GraBi = bita grains; GraBo = grains for porridge; GraCa = grains for engagement gifts; GraCh = grains for charity; GraCo = couscous grains; GraDo = Grains for late harvest donation ; GraEn = grains for social assistance; GraMa = grains for gift to marabouts; GraZa = grains for zakkat; Gre = grenier; Han = hangar; Jeuma = provision for bride; Grains; Lab = labdourou; Bed = beds; May = house; Malar =clay mixing; PanAl = panicle for livestock feed; Panfr = fresh pan for grillade; Pansè = dry pan for grilling; PanAu = Panicles for Alms; PanCh = panicles for charity; PanDo =panicles for donation in late harvest; PanEn = panicles for social assistance; PanMa = panicles for donation to marabouts; PanPa = panicles for donation to parents; PanPr = panicles for provision for primiparous women; PanZa = panicles for zakkat; Pât = paste; Por = portal; Pou = henhouse; Prife = grains for provision for primiparous woman; RacAl = spoiled for livestock feed; Rack = spit in ash for cooking; Sal = sala; Savmé = medical soap; Savno = black soap; Sék = Sékos; Sôk = Sôkou; SonAb = sound for livestock watering; SonAl = sound for livestock feed; SonBo = sound for boiled; SonCo = sound for couscous; SosKo = Sosso Komandi; Sou = souroundou; SubNa = ash as a substitute for natron; Was = Wassalé; Zor = Zori. HsAd = hausa adult; HsJe = hausa youth; HsVx = hausa old; KnAd = adult kanuri; KnJe = young kanuri; KnVx = old kanuri; PlhAd = adult fulani; PlhJe = young fulani; PlhVx = old fulani; TrgAd = adult Tuareg; TrgJe = young tuareg; TrVx = old tuareg; ZmAd = adult zarma-sonhrai; ZmJe = zarma-sonhrai young; ZmVx = old zarma-sonhrai

**Fig. 5:**
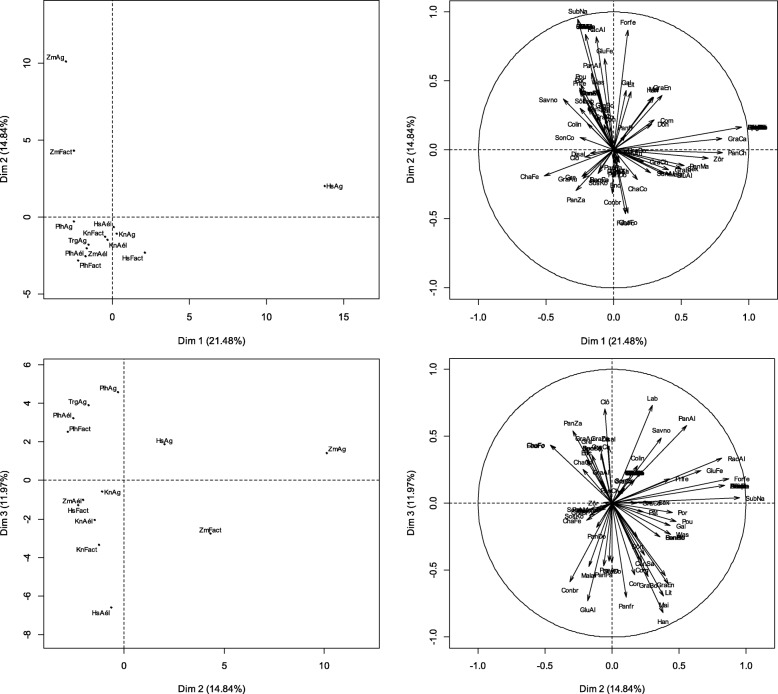
Factorial maps of the PCA describing the relationships between the specific uses of millet and the occupation-ethnic group factor. Note: HsAg = hausa farmer; HsAel = hausa agro-herder; HsFact = hausa others; KnAg = kanuri farmer; KnAel = kanuri agro-herder; KnFact = kanuri others; PlhAg = fulani farmer; PlhAel = fulani agro-herder; PlhFact = fulani others; TrgAg = tuareg farmer; ZmAg = zarma-sonhrai farmer; ZmAel = zarma-sonhrai agro-herder; ZmFact = zarma-sonhrai others

**Fig. 6:**
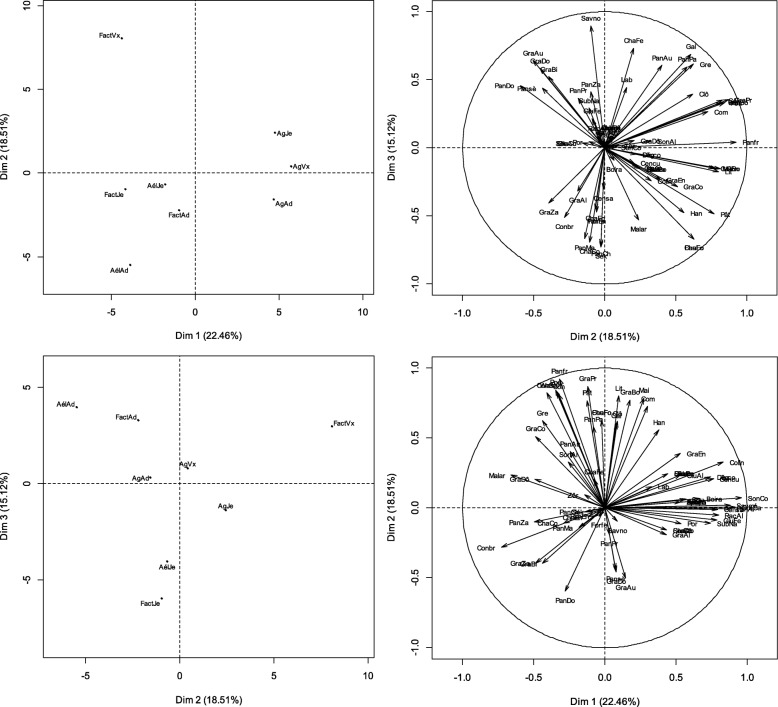
Factorial maps of the PCA describing the relationships between the specific uses of millet and the occupation-age factor. Note: AgAd = adult farmer; AgJe = young farmer; AgVx = old farmer; AelAd = agro-herder adult; AelJe = young Agro-herder; AelVx = Agro-herder old; FactAd = other adults; FactJe = other young people; FactVx = other old people
